# Enhancing Value-Based Care With a Walk-in Clinic: A Primary Care Provider Intervention to Decrease Low Acuity Emergency Department Overutilization

**DOI:** 10.7759/cureus.13284

**Published:** 2021-02-11

**Authors:** Derek J Baughman, Abdul Waheed, Muhammad N Khan, James M Nicholson

**Affiliations:** 1 Family Medicine, WellSpan Good Samaritan Hospital, Lebanon, USA; 2 Family Medicine, Wellspan Good Samaritan Hospital, Lebanon, USA; 3 Family and Community Medicine, Penn State University College of Medicine, Milton S. Hershey Medical Center, Hershey, USA

**Keywords:** emergency department utilization, family medicine, pcp, value based care, healthcare utilization, walk in clinic, pcp intervention, primary care medicine, health system research, health economics outcomes research

## Abstract

Background

Emergency department overutilization is a known contributor to the high per-capita healthcare cost in the United States. There is a knowledge gap regarding the substitution effect of walk-in clinic availability in primary care provider (PCP) offices and emergency department utilization (EDU). This study evaluates associations between PCP availability and EDU and analyzes the potential cost savings for health systems.

Methods

A retrospective cohort analysis compared low acuity EDU rates in established patients at a family medicine residency's PCP office before and after walk-in clinic implementation. The practice had 12 providers, 12 residents, and a patient panel of approximately 7,000-8,000. Inclusion criteria were met if patients were: (1) established with the PCP office, (2) had a low acuity emergency department (ED) visit (emergency index score level 4 or 5) *OR* had a walk-in clinic visit at the family practice. ED visits were tracked from January 2018 to January 2020 and encounters were compared numbers to pre and post-implementation of a walk-in clinic. Cost savings for comparable management was estimated with average price differences for low acuity encounters in the ED versus clinic.

Results

Over the two-year timeframe, there were 10,962 total visits to the ED by family practice patients, 4,250 of these visits were low acuity. Despite gross monthly increases of EDU from 2018-2020, after implementation of a walk-in clinic in 2019, rates of total EDU decreased by 1.5% and low acuity utilization rates also decreased. The average annual patient census nearly doubled from 5,763 to 8,042. T-tests confirmed statistical significance with p-values <0.05. Average low acuity ED visits ($437) cost 4.9 times more than comparable PCP office visits ($91). Managing 2,387 patients in the walk-in clinic resulted in an estimated annual cost savings of $825,902.

Conclusion

Extended walk-in availability in primary care offices provides non-ED capacity for low acuity management and might mitigate low acuity ED utilization while providing more cost-effective care. This study supports similarly described pre-hospital diversions in reducing ED over-utilization by increasing access to care. Higher levels of evidence are needed to establish causality.

## Introduction

The prevalence of emergency department overutilization for non-urgent care is common and is known to contribute to the high cost of US healthcare. Although the causes of non-urgent ED visits are multifactorial, the literature has described many of the social determinant factors well. Higher proportions of patients with material needs like employment, food, and housing insecurities utilize the ED [1-2]. The prevalence of low acuity ED visits is disproportionally high in Medicaid populations that have an increased number of social determinants and/or health illiteracy [3-4]. In addition, Medicaid populations are more than twice as likely to have a higher number of low acuity ED visits if they have one or more barriers, notably, difficulties in acute primary care appointment scheduling, transportation, or availability of office hours) [5]. Although there is evidence that the use of healthcare resources like case management or patient-centered medical homes (PCMHs) can decrease emergency department utilization (EDU) [6], healthcare utilization is a standalone contributing factor for increased EDU. For example, having >3 primary care provider (PCP) visits per year, prior hospitalizations, increased age, female gender, and the presence of a prior mental health diagnosis increases ED overutilization [7]. In addition, patients with higher ED utilization (EDU) have increased healthcare utilization overall, including outpatient and hospital admissions. These patients also have a 2.2-fold increased rate of mortality [8]. Patients with cancer, chronic non-cancer pain, and significant disabilities are also linked with EDU [4,9]. The reasons why patients feel the need to self-refer to the ED include, most commonly, the concern for personal health and the expectation of having these concerns evaluated. Other factors can include convenience, inaccessibility to primary care, lack of confidence in PCP, advice from others, and financial considerations [10].

Medical literature supports that some interventions might decrease lower acuity ED visits. Many studies have shown the mitigation of both low acuity EDU and cost of care via case management interventions [2,4,6,11-17]. The use of community health workers and patient education has also resulted in a significant benefit [2-3,6,16,18-21]. Other studies have analyzed various interventions that have been shown to decrease non-urgent EDU such as the creation of additional non-ED capacity (including walk-in hours or additional/extended business hours) [3,18,22]. The use of high-risk identifying prediction tools, interventions that followed patients beyond the referral process or simply having more involvement with the Patient-Centered Medical Home (PCMH) has shown to reduce cost and ED use [4,23-26]. Several studies demonstrate the cooperative effect of incorporating multiple interventions to decrease EDU [3,6,25]. While cost-sharing appears to reduce EDU [3], patient financial incentives seem to be not as effective [18,26]. Although some studies note equivocal rates of EDU reduction with diversion interventions [9,24], several studies have shown the success of pre-hospital diversions [27], including 911 telephone triaging and personally escorting patients from the ED to the clinic [18-19,21].

There is a paucity of analysis specifically on the use of walk-in clinic hours in PCP offices as an intervention for reducing low-acuity EDU. The purpose of this study was to evaluate the medical, financial, and logistical impact of providing walk-in clinic hours at a residency family practice site to manage acute medical conditions of a non-emergent nature and, in turn, decompress emergency department utilization in the health system. Considering the potential role of primary care interventions is an important step in addressing underlying causes of inflated per-capita health care costs since the impact of cost mitigation at the community level has conceivably exponential potential when applied to the state and national levels.

## Materials and methods

We performed a retrospective cohort study involving an urban PCP office in South Central Pennsylvania. The population in Lebanon, PA (a city of approximately 25,000), was predominantly Caucasian (58%) with significant Hispanic (25%) and Black (5%) minorities. These population patterns are similar to the distribution of race in the 2010 US census [28]. Our cohort was a sample of this population: established patients at WellSpan North 4th Street Family Medicine Office in Lebanon, PA (one of the Good Samaritan Hospital’s Family Medicine residency program PCP offices). “Established patient” was defined according to Medicare's definition - patients with a PCP visit within a historical three-year window [29]. Both our ED and PCP offices use the same electronic medical record (EMR) (Epic; Verona, WI), which allowed for the convenient generation of workbench reports for emergency room visit data by patients established at our practice. These data were obtained with IRB exemption for patients established at the practice from January 2015 to January 2020 in order to generate an established patient census from January 2018 to January 2020 (thus, for example, an established patient for the month of January in 2018 would have had a visit somewhere between January 2015 to January 2018 to meet the three-year window criteria).

Inclusion criteria for ED low acuity visits at Good Samaritan Hospital were according to the Agency for Healthcare Research and Quality Emergency Severity Index (ESI) scoring [30]. Low acuity was defined as ESI 4 or 5 and high acuity was defined as ESI 1-3. ED visits were captured from the affiliated Good Samaritan Hospital ED (only 0.8 miles from the North 4th Street PCP office). Walk-in clinic hours were on weekdays from 08:00 to 12:00 and monthly gross numbers were tracked in the EMR after their implementation in January 2019. Notably, only patients established at the practice (North 4th Street Family Medicine) were eligible to be seen in the walk-in clinic. These data were imported to Microsoft Excel (Microsoft Corporation, Redmond, WA) where 10,962 ED visits were analyzed from January 2018 to January 2020 alongside a monthly rolling census and total walk-in visit numbers (Table 1). In order to make a fair comparison between walk-in clinic hours and low acuity EDU, the low acuity visit numbers were adjusted to match the four-hour walk-in clinic hours timeframe of 08:00 to 12:00. This was termed “adjusted” EDU (essentially a sub-selection of the low acuity visits) where ED visits by clinic patients were only tabulated if the ED encounter was during the window 08:00-12:00).

**Table 1:** Summary of healthcare utilization data from January 2018 to January 2020 by established family practice patients LA: low acuity; HA: high acuity; EDU: emergency department utilization

Month-Year	Total High Acuity	Total Low Acuity	Adjusted LA EDU visits	Total ED visits	Rolling pts census	walk-in visits	HA EDU rate	LA EDU rate	Adjusted LA EDU rate	Total EDU rate	Walk-in visit rate
	ESI 1-3)	(ESI 4-5)		(established patients only)		(rates are per 100 patients)					
Jan-18	236	172	80	408	4423		5.3	3.9	1.8	9.2	
Feb-18	214	168	81	382	4705		4.5	3.6	1.7	8.1	
Mar-18	229	166	71	395	4998		4.6	3.3	1.4	7.9	
Apr-18	221	146	66	367	5211		4.2	2.8	1.3	7.0	
May-18	233	143	84	376	5460		4.3	2.6	1.5	6.9	
Jun-18	191	125	45	316	5691		3.4	2.2	0.8	5.6	
Jul-18	247	118	60	365	5937		4.2	2.0	1.0	6.1	
Aug-18	228	140	51	368	6133		3.7	2.3	0.8	6.0	
Sep-18	241	143	74	384	6351		3.8	2.3	1.2	6.0	
Oct-18	260	166	88	426	6547		4.0	2.5	1.3	6.5	
Nov-18	215	160	66	375	6759		3.2	2.4	1.0	5.5	
Dec-18	268	181	72	449	6945		3.9	2.6	1.0	6.5	
Jan-19	285	174	81	459	7118	41	4.0	2.4	1.1	6.4	0.6
Feb-19	255	169	80	424	7304	164	3.5	2.3	1.1	5.8	2.2
Mar-19	308	186	101	494	7478	157	4.1	2.5	1.4	6.6	2.1
Apr-19	322	184	91	506	7615	198	4.2	2.4	1.2	6.6	2.6
May-19	277	181	80	458	7761	183	3.6	2.3	1.0	5.9	2.4
Jun-19	286	179	71	465	7917	153	3.6	2.3	0.9	5.9	1.9
Jul-19	303	182	66	485	8033	180	3.8	2.3	0.8	6.0	2.2
Aug-19	321	169	86	490	8192	165	3.9	2.1	1.0	6.0	2.0
Sep-19	313	196	92	509	8356	226	3.7	2.3	1.1	6.1	2.7
Oct-19	286	186	93	472	8498	209	3.4	2.2	1.1	5.6	2.5
Nov-19	297	202	101	499	8655	211	3.4	2.3	1.2	5.8	2.4
Dec-19	310	180	78	490	8760	213	3.5	2.1	0.9	5.6	2.4
Jan-20	366	234	121	600	8855	287	4.1	2.6	1.4	6.8	3.2
Total pre-intervention	2,783	1,828	838	4,611							
average	232	152	69.8	384	5,763		4.1	2.7	1.2	6.8	
Total post-intervention	3,929	2,422	1,141	6,351		2,387					
average	302	186	87.8	489	8,042	184	3.8	2.3	1.1	6.1	2.3
Difference of averages pre and post-intervention	70.3	34.0	17.9	104.3	2278.4		-0.3	-0.4	-0.2	-0.7	

**Figure 1:**
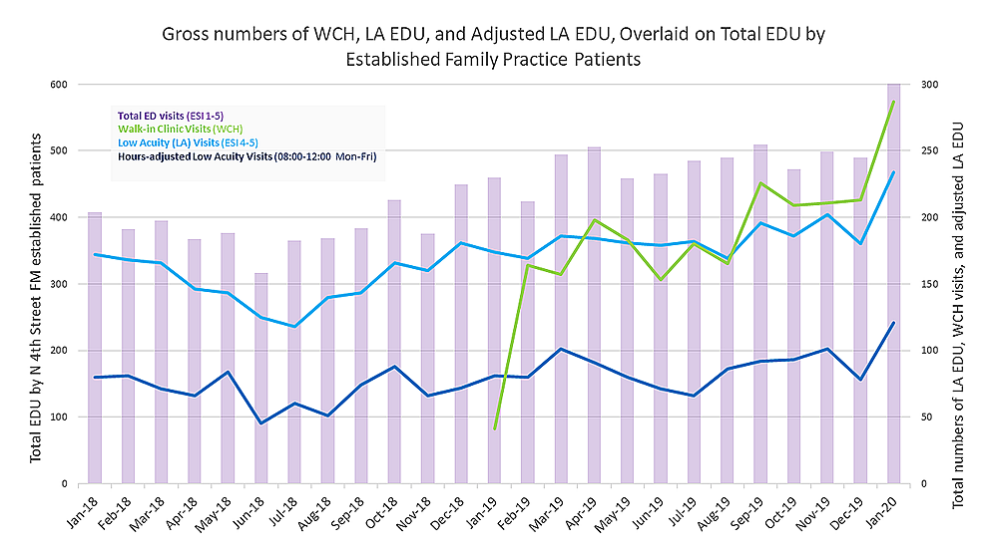
Gross numbers of WCH, LA EDU, and adjusted LA EDU, overlaid on total EDU established family practice patients The dark blue line is the adjusted low acuity visit hours to match the walk-in clinic hour timeframe; essentially, these are all the low acuity ED visits (light blue) that happened only between 0800 and 1200 hours. WCH: walk-in clinic hours; LA: low acuity; EDU: emergency department utilization

**Figure 2:**
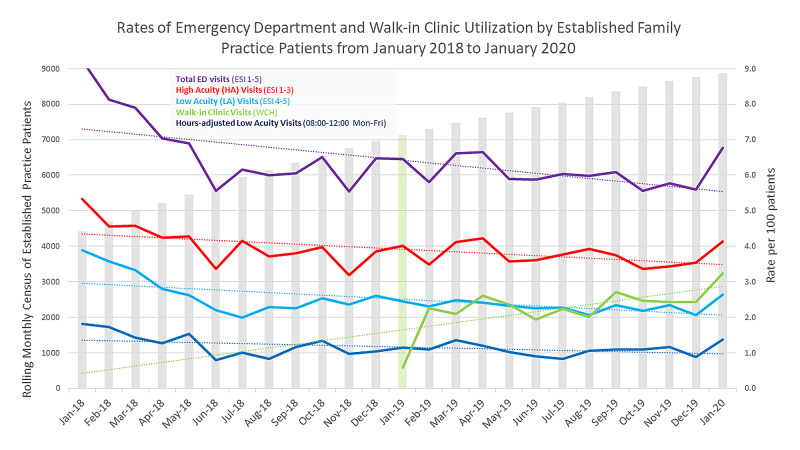
Rates of emergency department and walk-in clinic utilization by established family practice patients

Gross numbers of total EDU, high acuity, low acuity, and time adjusted low acuity EDU were plotted in Microsoft Excel (Figure 1). To calculate rates of EDU, two methods were used. First, gross low acuity EDU was divided by the total EDU, and second, the low acuity EDU was divided by the rolling established patient census (Figure 2). T-tests compared monthly data, pre- and post-implementation of walk-in clinic hours, for low and high acuity EDU, adjusted low acuity EDU, walk-in clinic hours visits, average and rolling census numbers (Table 2), and p-values of <0.05 measured statistical significance.

**Table 2:** Summary of statistical analysis (T-tests comparing mean visit data) and calculation of utilization rates LA: low acuity; EDU: emergency department utilization

2018	2019	2018-2019	p-value
Adjusted monthly LA EDU	69.8	87.8	1979	<0.05
Monthly avg LA (aLA)	152	186	170	<0.01
Monthly avg HA (aHA)	232	302	268	<0.01
Monthly avg EDU (aEDU)	384	489	438	<0.01
Monthly avg census (aC)	5,763	8,042	6948	<0.01
tLA/tEDU	39.6%	38.1%	-1.51%	
aLA/aEDU	39.6%	38.1%	-1.51%	
tLA/aC	31.7%	30.1%	-1.60%	
aLA/aC	2.6%	2.3%	-0.33%	

Finally, cost comparisons of the WellSpan Medical group were obtained via population health data from the Emig Research Center, WellSpan York Hospital (Table 3). Of note, the cost data were derived non-empirically and represent the average low acuity visit cost in the ED ($437) and corresponding average low acuity outpatient visit ($91). The total monthly visit numbers of low acuity ED visits and walk-in clinic visits were multiplied by $437 and $91, respectively. Monthly dollar amounts were summed for the estimation of annual spending in the ED versus the walk-in clinic. Estimated cost savings were determined by taking the difference of the average ED and ambulatory visit cost ($437-$91), multiplying the result by the total number of patients seen in the walk-in clinic.

**Table 3:** Cost-savings projection of walk-in clinic implementation 1. The average cost of a Good Samaritan Hospital emergency Level 1 visit (CPT code: 99281) was $437. 2. The average cost for a WellSpan Med Group established patient for a Tier 1 visit was either $68 (CPT code: 99212) or $114 (CPT code: 99213). The average of $68 and $114 is $91. 3. Calculation for the average cost difference between a WCH and LA ED visit: avg Level 1 ED visit / avg ambulatory visit 4. Calculation for estimated cost savings in 2019 due to WCH: total WCH visits 2019 x ($437-$91) Source: Epic EMR workbench reports with Emig research center average billing cost data for low acuity visits. Cost estimations from the WellSpan medical group are average billing data for equivalent low acuity visit costs in the ED vs the ambulatory setting. WCH: walk-in clinic hours; LA: low acuity; EDU: emergency department utilization; CPT: current procedural terminology

Date	ESI 4	ESI 5	Total LA ED visits	Average level 1 ED visit ($437)^1^	Total WCH visits	Ambulatory tier 1 ($91)^2^
01 2018	160	12	172	$75,164		
02 2018	164	4	168	$73,416		
03 2018	148	18	166	$72,542		
04 2018	130	16	146	$63,802		
05 2018	123	20	143	$62,491		
06 2018	112	13	125	$54,625		
07 2018	99	19	118	$51,566		
08 2018	125	15	140	$61,180		
09 2018	128	15	143	$62,491		
10 2018	149	17	166	$72,542		
11 2018	144	16	160	$69,920		
12 2018	169	12	181	$79,097		
01 2019	158	16	174	$76,038	41	$3,731
02 2019	155	14	169	$73,853	164	$14,924
03 2019	176	10	186	$81,282	157	$14,287
04 2019	164	20	184	$80,408	198	$18,018
05 2019	162	19	181	$79,097	183	$16,653
06 2019	173	6	179	$78,223	153	$13,923
07 2019	169	13	182	$79,534	180	$16,380
08 2019	159	10	169	$73,853	165	$15,015
09 2019	180	16	196	$85,652	226	$20,566
10 2019	169	17	186	$81,282	209	$19,019
11 2019	185	17	202	$88,274	211	$19,201
12 2019	166	14	180	$78,660	213	$19,383
01 2020	221	13	234	$102,258	287	$26,117
Total 2018-2019	3888	362	4250	$1,857,250	2387	$217,217
average 2018	138	15	152	$66,570		
total 2019	2237	185	2422	$1,058,414	2387	$217,217
average 2019	172	14	186	$81,416	184	$16,709
	Average cost difference between WCH & LA ED visit^3^				4.9	
	Estimated cost savings in 2019 due to WCH^4^				$825,902	

## Results

The established office census nearly doubled and the average monthly census increased from 5,763 in 2018 to 8,042 in 2019 (Table 1). The gross numbers of total EDU (both high and low acuity EDU) increased from January 2018 to January 2020 (Figure 1), with an average monthly total EDU (tEDU) increasing from 384 to 480, respectively. Gross low acuity EDU increased by an average difference of 34 patients per month; walk-in clinic hours adjusted low acuity EDU numbers increased on average by 10 patients per month, however, calculated rates of low acuity EDU decreased (when both denominators of tEDU and per 100 patients were used, Figure 2) and total low acuity EDU decreased by 1.5% (Table 2). Rates had a negative correlation coefficient (r2 = 0.43).

Walk-in clinic hours visit numbers eventually exceeded total low acuity ED visits after September 2019, however, from January 2019 to January 2020, the average difference in visits per day between walk-in clinic hours and low acuity total EDU was only 2.7 patients. On average, in 2019, there were 95 more patients per month seen in walk-in clinic hours than seen at hours-adjusted low acuity EDU (monthly avg walk-in clinic hours = 184, avg hours-adjusted low acuity EDU = 88). A comparison of monthly mean visits from January 2018 to January 2020 between EDU and walk-in clinic hours were statistically significant (Appendix).

The average total estimated monthly costs for EDU and walk-in clinic hours for 2019 were $81,416 and $16,709 respectively (Table 3). Thus, in 2019, an estimated average low acuity visit in the ambulatory setting was approximately 1/5th (20.4%) as expensive as a comparable low acuity ED visit. USD 825,902 was the projected cost savings by seeing 2,387 patients in the clinic (instead of the ED).

## Discussion

First, our analysis demonstrates a statistically significant association between increased availability of walk-in clinic hours and lower rates of low acuity EDU in established patients. Although not causative, the association of provider availability and decreased utilization is consistent with recent literature describing the creation of additional non-ED capacity to decrease EDU [3,18,22]. This has also been shown to increase follow-up at PCMHs [4,24-25].

Second, low acuity care in the ambulatory setting was more cost-effective, and at WellSpan, it was about 1/5th the cost. Despite the gross simplification, conceivably, without adding a single physician or staff member, the North 4th Street clinic saved $825,902 by seeing low acuity patients in the walk-in clinic (Table 3). Explained hypothetically, if the 2387 patients seen in the walk-in clinic in 2019 had gone instead to the ED, that estimated cost would be $437 x 2387 or $1,043,119 (when in actuality, the comparative annual ambulatory total cost was $217,217). The authors recognize this is not a formal cost-effective analysis (as access to payer data was not available), nonetheless, it is reasonable to conclude the frugality of ambulatory management given comparable management severity.

Third, it took only a small amount of time for walk-in clinic numbers to exceed low acuity EDU numbers. After the implementation of walk-in clinic hours, it took only six months for the number of walk-in clinic visits to exceed the total number of low acuity EDU visits (Figure 1). This comparison becomes even more meaningful when hours of EDU are adjusted to match low acuity EDU visit numbers to the 08:00-12:00 walk-in clinic hours window. When only 08:00-12:00 low acuity visits were counted, the comparison to walk-in numbers is dramatic: more than double the number of patients were seen in walk-in just one month after implementation.

Fourth, facilitating lower acuity patient visits in the clinic mitigates the consumption of ED physician resources and frees up the ED for truly emergent patients. We describe this as ‘decompression’ of the ED. Decompression is an indirect effect of managing illness acuity in the appropriate clinical setting. The result of this appropriate alternative - decompressive outpatient management - enables ED providers to focus their attention on triaging the sickest patients while ensuring appropriate resources are available for urgent and emergent cases.

Finally, our study demonstrated a positive association between walk-in clinic availability and our practice census. We know that PCP follow-up [3-4,6,25] and office education [2-3,6,16,19,21] are investments that ultimately enhance value-based care by reducing unnecessary EDU. Thus, investing in PCP access via interventions like walk-in clinic hours has the feed-forward effect of enhancing PCP follow-up and the subsequent office education thereof. Accordingly, there was likely a delayed but potentially exponential effect of non-ED capacity that contributed to the doubling of our office census in 2019. Especially for patients with complex care needs, PCMH has consistently shown to both strengthen itself and mitigate known determinants to reduce cost and EDU [3,15]. As offering alternative treatment options has demonstrated a diversion of low acuity visits from the ED to the PCP, there is a potential for augmenting further public health benefits such as preventive care [2,19,21]. We are interested in continuing to follow practice data to support this hypothesis.

Although there is literature on ED utilization and super-utilization, the data are limited when it comes to connecting the dots between clinic patient panels and their respective EDU. Our study specifically investigated this concept and came across a challenge of defining the meaningful denominator and numerator to reflect the outcome. We knew that in the rate equation (low acuity EDU/total established patients), ‘low acuity EDU’ was the obvious numerator. The choice for denominator was a trial and error process but we ultimately chose ‘Total established patients’ to reflect the impact of walk-in clinic hours on building patient census (as this is the desired effect on a PCMH in implementing walk-in clinic hours). However, we recognize that using the total number of established patients may blur the association of decreasing low acuity EDU rates by “diluting” the numerator. Are walk-in clinic hours causing a decrease in the rate of EDU by providing an appropriate, yet more affordable, alternative venue? Or, are walk-in clinic hours causing an increasing rolling patient census that confounds the association with low acuity EDU rate reduction (i.e. a dilution of the numerator)? This is difficult to conclude, but what provides support to the former (decreasing the rate of low acuity EDU) is considering the correlation plots. When walk-in clinic hours are similarly converted to a rate (walk-in clinic hours/total rolling census), the correlation is positive (as expected with increasing numbers of walk-in visits). Conversely, even when low acuity EDU is adjusted to the 08:00-12:00 timeframe (adjusted low acuity EDU/total established patients), the correlation remains negative (decreased numbers of low acuity EDU in comparison to total established patients).

We recognize several limitations. Notable is the analysis of cost. It is difficult to cross-tabulate payment data for ESI 4-5 visits. After consulting payment experts in our health system, the authors felt that average visit cost data was an appropriate equivalent for financially quantifying low acuity EDU. More importantly, we feel this highlights the difficulty of obtaining such numbers since what is billed and what is paid is quite variable and is very hard to quantify (even for billing departments at the health system level). We recognize this limits the strength of cost-association, but does not promote the ignorance of cost-effective claims made in the discussion. We also recognize that our ability to draw conclusions regarding social determinants of health, notably, access to care, is also limited by minimal demographic data. Social factors (age, race/ethnicity, payer type, comorbid conditions, and other risk factors) could better define the population and augment value-based care (VBC) arguments.

In the future, we plan to study how billing data acquisition highlights larger issues in certain reimbursement models within health systems. Despite proving a clear cost-benefit to society, there is actually a disincentive for hospitals operating in a fee for service (FFS) model to endorse walk-in clinic hours as a cost mitigation strategy (since the reimbursement relies on what is billed, not recuperated savings). In FFS, there is less incentive for encouraging patients to use ambulatory venues (like walk-in clinic hours) for low acuity problems when the hospital can hypothetically bill nearly five times more by seeing the same patient in the ED. However, steering policy efforts toward supporting an ambulatory focus for low acuity EDU in FFS models may be soon obsolete since most modern health systems are progressing toward VBC. Thus, for health systems operating in a VBC model, it is favorable to increase the use of ambulatory care for low acuity visits to offload overutilization of the ED. Moreover, with VBC, the per capita savings with ambulatory management is a sensible financial incentive to support the model. It is more cost-effective, enhances the opportunity for preventive care, and increases follow-up at PCHMs. 

## Conclusions

The implementation of walk-in clinic hours was associated with decreased rates of low acuity ED utilization in patients at our family medicine practice. In our health system, a low acuity ambulatory visit costs nearly 1/5th that of a comparable ED visit, demonstrating the potential for per capita cost reduction. These findings support the medical literature and may lead to improved follow-up within PCMHs. As value-based care is the progression of modern healthcare, efforts to facilitate increased ambulatory management for low acuity care is a financial incentive. Future research should measure the intervention effect across multiple healthcare sites where randomization to offering or not offering walk-in clinic hours could measure relative changes in EDU to conclude causality rather than association. Such evidence could support executive decisions to increase the implementation of non-ED capacity (especially walk-in clinics at PCP sites) for low acuity care in health systems to enhance the model of value-based care.

## Appendices

**Table 4:** T-tests comparisons of utilization data tLA: total low acuity; aLA: average low acuity; tHA: total high acuity; aHA: average high acuity; tEDU: total emergency department utilization; aEDU: average emergency department utilization

T-Test: Two-Sample Assuming Equal Variances	Rolling Census	
	2018	2019
Mean	5763.333333	8041.692308
Variance	674418.2424	322900.3974
Observations	12	13
Pooled Variance	491017.6276	
Hypothesized Mean Difference	0	
df	23	
t Stat	-8.122054603	
P(T<=t) one-tail	1.65233E-08	
t Critical one-tail	1.713871528	
P(T<=t) two-tail	3.30466E-08	
t Critical two-tail	2.06865761	

T-Test: Two-Sample Assuming Equal Variances	Total EDU	
	2018	2019
Mean	384.25	488.5384615
Variance	1124.022727	1675.102564
Observations	12	13
Pooled Variance	1411.542642	
Hypothesized Mean Difference	0	
df	23	
t Stat	-6.93396355	
P(T<=t) one-tail	2.27525E-07	
t Critical one-tail	1.713871528	
P(T<=t) two-tail	0.00000046	
t Critical two-tail	2.06865761	

t-Test: Two-Sample Assuming Equal Variances	HA EDU	
	2018	2019
Mean	231.9166667	302.2307692
Variance	440.2651515	728.1923077
Observations	12	13
Pooled Variance	590.4880156	
Hypothesized Mean Difference	0	
df	23	
t Stat	-7.228183829	
P(T<=t) one-tail	1.16801E-07	
t Critical one-tail	1.713871528	
P(T<=t) two-tail	2.33603E-07	
t Critical two-tail	2.06865761	

t-Test: Two-Sample Assuming Equal Variances	LA EDU	
	2018	2019
Mean	152.3333333	186.3076923
Variance	379.8787879	292.5641026
Observations	12	13
Pooled Variance	334.3232999	
Hypothesized Mean Difference	0	
df	23	
t Stat	-4.641520188	
P(T<=t) one-tail	5.687E-05	
t Critical one-tail	1.713871528	
P(T<=t) two-tail	0.00011374	
t Critical two-tail	2.06865761	

t-Test: Two-Sample Assuming Equal Variances	Adjusted LA EDU	
	2018	2019
Mean	69.83333333	85
Variance	170.8787879	121.2727273
Observations	12	12
Pooled Variance	146.0757576	
Hypothesized Mean Difference	0	
df	22	
t Stat	-3.073807695	
P(T<=t) one-tail	0.002777339	
t Critical one-tail	1.717144374	
P(T<=t) two-tail	0.005554679	
t Critical two-tail	2.073873068	
